# cljam: a library for handling DNA sequence alignment/map (SAM) with parallel processing

**DOI:** 10.1186/s13029-016-0058-6

**Published:** 2016-08-17

**Authors:** Toshiki Takeuchi, Atsuo Yamada, Takashi Aoki, Kunihiro Nishimura

**Affiliations:** Xcoo, Inc., 4-2-5, Hongo, Bunkyo-ku, Tokyo, Japan

**Keywords:** Next-generation sequencing, DNA, Parallel processing, Clojure, SAM, BAM

## Abstract

**Background:**

Next-generation sequencing can determine DNA bases and the results of sequence alignments are generally stored in files in the Sequence Alignment/Map (SAM) format and the compressed binary version (BAM) of it. SAMtools is a typical tool for dealing with files in the SAM/BAM format. SAMtools has various functions, including detection of variants, visualization of alignments, indexing, extraction of parts of the data and loci, and conversion of file formats. It is written in C and can execute fast. However, SAMtools requires an additional implementation to be used in parallel with, for example, OpenMP (Open Multi-Processing) libraries. For the accumulation of next-generation sequencing data, a simple parallelization program, which can support cloud and PC cluster environments, is required.

**Results:**

We have developed cljam using the Clojure programming language, which simplifies parallel programming, to handle SAM/BAM data. Cljam can run in a Java runtime environment (e.g., Windows, Linux, Mac OS X) with Clojure.

**Conclusions:**

Cljam can process and analyze SAM/BAM files in parallel and at high speed. The execution time with cljam is almost the same as with SAMtools. The cljam code is written in Clojure and has fewer lines than other similar tools.

## Background

Next-generation sequencing (NGS) technologies have allowed DNA sequences to be generated very fast and in parallel. Complete DNA sequences can be obtained by statistical analysis of the raw data from the sequencers. As a result, tools for data analysis and interpretation of the sequencing results are in high demand. For maximum efficiency, data should be processed in parallel and with high speed considering the accumulation speed and size of NGS data. A lightweight program that can deal with NGS data in parallel is required.

Most NGS sequencers generate hundreds of millions of short sequence reads for each DNA or RNA sample. These short read data are small pieces of DNA sequence bases. The DNA and RNA sequence data are saved mainly in FASTQ format, which is a text-based format for sequences and their quality scores. Typically, FASTQ files contain about 300 million reads that are about 200-300 nucleotides long. The short reads in FASTQ files are generally mapped and aligned to a reference genome with alignment mapping tools such as BWA [1] and Bowtie [2]. The alignment data are stored mainly in Sequence Alignment/Map (SAM) format files, which are tab-delimited text files. BAM is the compressed binary version of the SAM format. BAM uses BGZF (Blocked GNU Zip Format) compression and can support indexes to achieve fast random access by generating BAM index (BAI) files.

SAMtools [3, 4] is written in the C programming language and uses SAM/BAM files. It has various functions for manipulating SAM/BAM files, such as viewing, sorting, indexing, and pileup. The ‘index’ command creates a BAI file for fast random access to the original BAM file. Counting the overlapping short read bases at a specified location is called pileup. The ‘mpileup’ command executes pileup and outputs the results in text format, which is useful for visualizing genome histograms and for detecting variants/insertions/deletions in a genome sequence.

SAM/BAM utilities are also available in other programming languages. Picard [5] is a Java-based command-line utility for manipulating high-throughput sequencing data and formats such as SAM and BAM. Because of their performance, some lightweight languages have been used to wrap SAMtools. For example, pysam [6] is a lightweight wrapper of SAMtools C-API written in the Python programming language, and bio-samtools [7, 8] is a Ruby language interface to SAMtools.

## Implementation

### The Clojure programming language

Clojure is a lightweight programming language that is favored for huge data analysis with parallel processing [9]. It is a functional programming language and is a dialect of Lisp. Clojure runs on the Java Virtual Machine, which includes Windows, Mac OS, and Linux environments. It is based on Java, which allows Java libraries to be used. Genome sequence analyses processes can be written simply because Clojure provides many convenient functions for manipulating list data. Moreover, immutability is the center of Clojure’s design policy so that parallel processing can be used efficiently.

Clojure has been used to code some bioinformatics tools. For example, BioClojure [10] is a convenient library for bioinformatics under the open source Bio* projects. It consists of parsers for various kinds of file formats (UniProtXML, Genbank XML, FASTA, and FASTQ), and wrappers of data analysis programs (BLAST, SignalP, TMHMM, and InterProScan). However, BioClojure does not have functions for SAM/BAM manipulation and is not fully implemented in Clojure. The CONNJUR-Sandbox source [11] contains examples of the visualization of protein structures using PDB data in Clojure and the prediction of neighboring amino acids with Support Vector Machine algorithms named Clojure Amino Acid Predictor.

### cljam

Here, we describe cljam, a SAM/BAM manipulating library written in Clojure. With cljam, we aimed to provide a much more simple source code than SAMtools that is equal in performance and can work in a Clojure ecosystem.

Cljam is not a SAMtools wrapper. It does not use an external application programming interface (API) such as SAMtools and Picard for simple and high maintainable codebase. Programs in Clojure are not as fast on a single thread, but because of its parallel processing functions it can be easily sped up. Cljam supports multithreaded processing in high-cost features such as BAM indexing and pileup. Parts of File I/O are written in Java because of high-speed processing. Cljam uses an open-source compression library for BGZF, named bgzf4j [12], which was developed by the authors of this paper.

Cljam has the following functions:

- Reading and writing SAM/BAM/FASTQ
- Converting SAM/BAM
- Normalization
- Sorting
- Indexing BAM
- Pileup
- Indexing FASTA

## Results and discussion

### Using cljam: a brief tutorial

Here are examples of interacting with SAM/BAM files using cljam. More information on usage and specific functions is provided in the readme file and https://chrovis.github.io/cljam/.

#### Installation

Cljam is available as a Clojure library at Leiningen, a popular build tool for Clojure projects. The following statement should be added to a Leiningen configuration.



Leiningen automatically downloads the Java Archive of cljam and resolves its dependency in a project. Then, cljam functions can be used in the code.

#### Reading a SAM/BAM file

Cljam provides a file reader and a namespace including various I/O functions to read a SAM/BAM file. The following code opens a BAM file and retrieves the first five alignments, where pnext, tlen, flag, qname, and rname indicate the potision of the mate/next read, observed template length, bitwise flag, query template name, and reference sequence name, respectively, based on the SAM format [13].



#### Sorting a SAM/BAM file

A SAM/BAM file can be sorted by chromosomal coordinates or reference name using functions in the ‘cljam.sorter.’ For example, to create a BAM file sorted by chromosomal coordinates,



In this case, the input and output files are file.bam and sorted.bam, respectively.

#### Indexing a BAM file

The ‘cljam.bam-indexer’ has functions for indexing a BAM file. The following code creates a BAI file from a BAM file.



#### Getting pileup information

The ‘cljam.pileup’ provides pileup and mpileup functions equivalent to those of SAMtools. For example, to get simple pileup of the first 10 genomic positions of chr1 reference,



#### Command line interface

The command line interface of cljam provides an additional feature to quickly check its functions. For example, the following command displays contents of a SAM file including header information.



### Performance of indexing and pileup

We conducted timing measurement experiments to determine the performance of BAM indexing and pileup under a changing number of thread conditions: 1, 2, 4, 8, and 12 threads with cljam (v0.1.3), SAMtools (v1.2) (single thread), and Picard (v1.134) (single thread). We used a BAM file (about 13.2GB) from the 1000 Genomes Project [14]. The machine specifications were CPU: Intel Core i7-4930K @ 3.40 GHz, 12 MB L2 cache, 12 cores (6 real cores & HT), 64 GB RAM, and SSD storage.

**Fig. 1:**
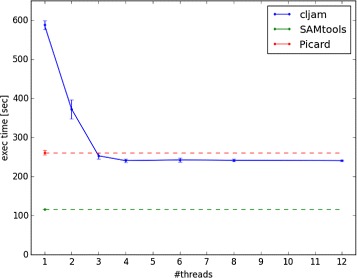
Execution time of indexing. The green dashed line indicates SAMtools and the red dashed line indicates Picard under single thread conditions because they cannot be run using multithreaded processing. The error bar shows the standard deviation of the result

**Fig. 2:**
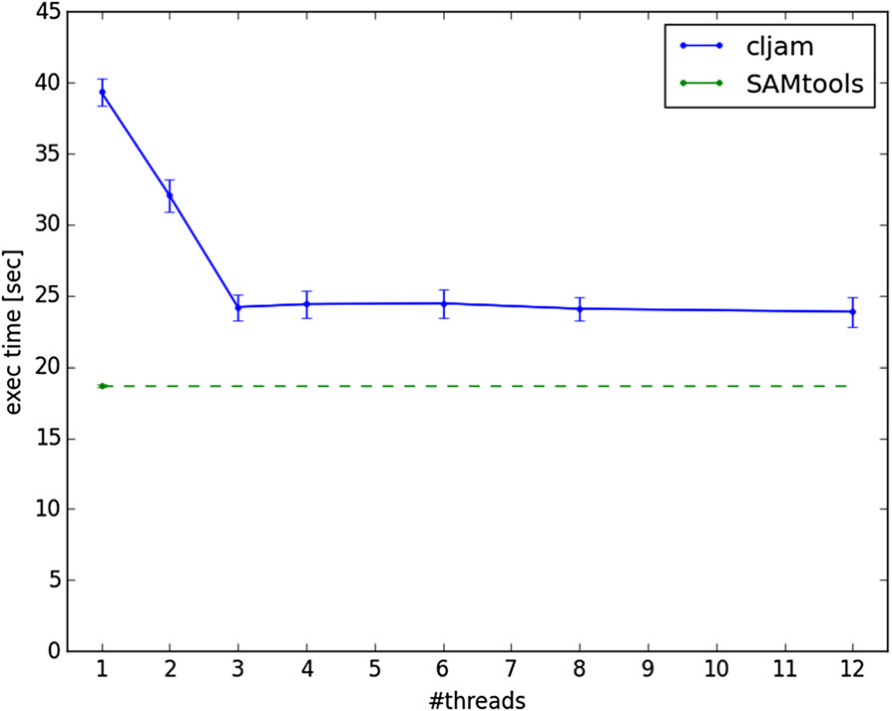
Execution time of pileup. The green dashed line indicates SAMtools under a single thread condition because it cannot be run using multithreaded processing. The error bar shows the standard deviation of the result

The results for indexing and pileup are shown in Figs. 1 and 2, respectively. Each condition was measured 10 times and the average time of the 10 trials was plotted.

The results indicate that the execution times for cljam were getting shorter until the 4 thread condition in indexing and 3 thread in pileup. However, the execution times under the conditions of above 6 threads in indexing and 4 threads in pileup were almost same. We believe there may be an overhead of the file I/O when reading BAM files; the performance does not improve in parallel conditions. The execution time of pileup in cljam with the 3 thread condition was 1.3 times longer than with SAMtools, which can be considered as almost the same performance.

### Code metrics

Code readability and maintainability are more important than optimization of code under our software development environment, which uses recent high-speed and multi-core CPU technologies. Thus, we used CLOC [15] to measure logical LOC (lines of code) of source codes of cljam, SAMtools, and Picard. The results indicate that the LOC of cljam was about 1/4 that of SAMtools and 1/9 that of Picard, as shown in Table 1. These three programs do not have all the same functions; thus, they cannot be compared only using LOC. Cljam has been implemented simply in Clojure with parallel programming with multi-core processors and with the focus on readability and maintainability.

**Table 1:** Measurement of LOC

Language	Files	Blank	Comment	Code
cljam (Clojure)	46	466	165	3264
SAMtools (C/C++)	53	1606	2403	11619
Picard (Java)	290	6409	11835	28322

## Conclusions

We have developed cljam as an open-source software using Clojure, which is a functional programming language that works on the Java Virtual Machine. Cljam can process and analyze SAM/BAM files in parallel and at high speed. The execution time with cljam is almost the same as with SAMtools. The Clojure code of cljam has fewer lines and an equivalent performance compared with SAMtools and Picard, which are similar tools.

## Availability and requirements

**Project name:** cljam**Project home page:**https://github.com/chrovis/cljam**Operating system(s):** Platform independent**Programming language:** Clojure**Other requirements:** none**License:** The Apache License, Version 2.0**Any restrictions to use by non-academics:** none

## Abbreviations

BAI: BAM index
BGZF: Blocked GNU zip format
LOC: Lines of code
NGS: Next generation sequencing
SAM: Sequence alignment/map
